# Resource-dependent attenuation of species interactions during bacterial succession

**DOI:** 10.1038/ismej.2016.11

**Published:** 2016-02-19

**Authors:** Damian W Rivett, Thomas Scheuerl, Christopher T Culbert, Shorok B Mombrikotb, Emma Johnstone, Timothy G Barraclough, Thomas Bell

**Affiliations:** 1 Department of Life Sciences, Imperial College London, Ascot, Berkshire, UK

## Abstract

Bacterial communities are vital for many economically and ecologically important processes. The role of bacterial community composition in determining ecosystem functioning depends critically on interactions among bacterial taxa. Several studies have shown that, despite a predominance of negative interactions in communities, bacteria are able to display positive interactions given the appropriate evolutionary or ecological conditions. We were interested in how interspecific interactions develop over time in a naturalistic setting of low resource supply rates. We assembled aquatic bacterial communities in microcosms and assayed the productivity (respiration and growth) and substrate degradation while tracking community composition. The results demonstrated that while bacterial communities displayed strongly negative interactions during the early phase of colonisation and acclimatisation to novel biotic and abiotic factors, this antagonism declined over time towards a more neutral state. This was associated with a shift from use of labile substrates in early succession to use of recalcitrant substrates later in succession, confirming a crucial role of resource dynamics in linking interspecific interactions with ecosystem functioning.

## Introduction

Bacterial communities are key components of most ecosystems, often having a critical role in a wide range of ecologically and economically important functional processes. Of particular interest is their role during decomposition, especially in breaking down complex organic molecules and recycling organic substrates to support primary production (Kardol *et al.*, 2010; McGuire and Treseder, 2010). Prior studies using simplified bacterial communities have shown that proxies of decomposition rate are sensitive to changes in diversity and composition (Bell *et al.*, 2005; Langenheder *et al.*, 2012). There is therefore considerable interest in understanding the generality of these results, the factors that govern variability in the link between community structure and function and the applicability of these simplified communities to natural ecosystems. Here, we explicitly test how dynamic interspecific interactions change over time, and how these interactions are linked to resource utilisation community productivity.

Prior studies have isolated bacteria from natural environments, and used these isolates to manipulate the diversity and composition of communities in laboratory microcosms (Wohl *et al.*, 2004; Bell *et al.*, 2005). Experimental studies have generally confirmed that there is an increasing, decelerating relationship between the taxonomic richness of the community and proxies of decomposition (for example, community respiration, activity, population growth) such that the rapid increase in functioning at low levels of species richness tails off at high levels of richness (Bell *et al.*, 2005; Gravel *et al.*, 2011; Langenheder *et al.*, 2012; Fiegna *et al.*, 2015). Just as for studies using multicellular organisms, the shape of the relationship has been explained in terms of the type of interspecific interactions (for example, competition, facilitation) in the community (Loreau and Hector, 2001). Therefore, a linear relationship between species richness and ecosystem functioning would be expected if species do not interact, whereas negative (for example, functional redundancy, resulting in competition) or positive (for example, cross-feeding) interactions would create deviations from linearity (Bell *et al.*, 2005; Gravel *et al.*, 2011; Lawrence *et al.*, 2012).

It is difficult in practice to measure microbial interactions directly, thus studies have usually looked at whether adding species to a community will harm or benefit ecosystem functioning (Bell *et al.*, 2005; Foster and Bell, 2012; Lawrence *et al.*, 2012). In the simplest case, net interactions can be assessed by comparing ecosystem functioning in mixtures of species to ecosystem functioning in pure cultures. In long-term bacterial microcosm experiments, we can test the null hypothesis that species are not interacting (that is, independent impacts on functioning) by comparing functioning of mixtures of species to the combined functioning of the constituent monocultures. Any deviation from this null hypothesis is consistent with either net negative (mixture functioning is less than predicted from the constituent monocultures) or positive (mixture functioning is greater than the combined monocultures) interactions (Fiegna *et al.*, 2015). Although the best method for predicting mixture functioning from pure cultures is not resolved, studies using bacterial communities constructed from culturable isolates have found that interactions tend to be negative (mixture functioning is less than the sum of monocultures; Foster and Bell, 2012), resulting in the observed decelerating relationship between species richness and ecosystem functioning. Interspecific interactions, however, are dynamic over short evolutionary time scales (~100 generations) (Lawrence *et al.*, 2012; Fiegna *et al.*, 2015), and can change rapidly when subjected to selection (Gravel *et al.*, 2011). For example, interactions can depend on the community context (Lawrence *et al.*, 2012), including the diversity of the surrounding community (Fiegna *et al.*, 2015).

There is much interest in understanding the temporal dynamics of bacterial communities in both applied and environmental microbiology. For example, if interspecific interactions follow a predictable trajectory during succession, it might be possible to understand and predict community development in newly created communities, such as those colonising newly formed leaves or during disease development. Prior studies have compared interactions in different environments (for example, Zhang *et al.*, 2009), but have not tracked how interspecific interactions develop over time. Here, we were interested in how interactions change during the succession and early development of a bacterial community encountering a novel environment. Although several studies have investigated colonisation and long-term succession of bacteria within a community in several environments (Dang and Lovell, 2000; Sulaiman *et al.*, 2014; Vallès *et al.*, 2014), these studies simply tracked changes in community composition.

We predicted that there would be a reduction in the strength of interactions during succession. First, strong interactions are likely to be attenuated during early successional stages because of competitive exclusion (Kokkoris *et al.*, 1999) and because communities containing strongly interacting species tend to be unstable (McCann *et al.*, 1998; Coyte *et al.*, 2015). Second, species' phenotypes might change (via evolution or phenotypic plasticity) to reduce strong interactions and increase fitness. Third, labile substrates are likely to be used early in succession, leaving primarily recalcitrant substrates during later successional stages (Eilers *et al.*, 2010). If degradation of recalcitrant substrates relies on more specialised feeding modes or on energy-expensive breakdown pathways, we expected negative interactions to dissipate during succession in the transition from communities that use labile substrates to communities that use recalcitrant substrates (Gravel *et al.*, 2011). In addition, if recalcitrant substrates are efficiently metabolised by only some members of the species pool (Eisenhauer *et al.*, 2013), then the overall decomposition rate should be correlated with diversity during late succession, but not during early succession. If, however, the recalcitrant resources can be efficiently metabolised by bacteria that can use many nutrient sources equally, then there would be little or no effect of richness at any successional point.

We tested these ideas in a simple microcosm system with low nutrient replacement that mimics conditions in water-filled beech treeholes in nature, which have been used as model aquatic ecosystems (Bell *et al.*, 2005). We constructed bacterial communities of differing levels of species richness from a pool of 16 environmental isolates. We tracked the communities over 49 days while measuring community-wide respiration along with their ability to metabolise labile and recalcitrant carbon substrates using enzymatic assays. We estimated interspecific interactions by comparing monoculture functioning to functioning of mixtures of species.

## Materials and methods

### Bacterial isolates and experimental setup

All of the 16 bacterial isolates included in this study were cultured from rain-filled depressions in the roots of beech trees (*Fagus sylvatica*) (Bell *et al.*, 2005). Isolation was undertaken following incubation on R2A agar (Sigma-Aldrich, Gillingham, UK) at 22 °C for 3 days with purified isolates stored in the ProtectTube Cryobead Systems (Technical Consultants Ltd, Heywood, UK). Bacterial isolates were selected based on their abundance within the treehole culture collection and their ability to produce three enzymes that are needed to degrade leaf-associated biological matter for nutrients. The isolates were separated into a single cluster (*n*=15) with a single outlier (Supplementary Figure S1), using a principal component analysis; this separation was based on the favoured substrate of the isolates. The identities of the isolates (Supplementary Table S1) were confirmed through 16S rRNA gene sequencing (all sequences were deposited in GenBank under the accession numbers: KT248518–KT248533) as members of the genera *Acinetobacter* (*n*=1), *Bacillus* (*n*=1), *Epilithomonas* (*n*=1), *Flavobacterium* (*n*=1), *Microbacterium* (*n*=2), *Pedobacter* (*n*=1), *Pseudomonas* (*n*=7), *Sphingomonas* (*n*=1) and *Staphylococcus* (*n*=1). The over-representation of *Pseudomonas* in the experiment (43% of all isolates in the pool of 16) was because they were the dominant genus in the culture collection (30% relative abundance). All of the isolates were initially grown in beech-leaf tea, prepared as described by Lawrence *et al.* (2012) supplemented with 200 μg ml^−1^ of an anti-fungal agent (cycloheximide; Sigma-Aldrich), for 4 days before the start of the experiment. The abundance of cells was measured using flow cytometry (see below). All isolates were diluted to an abundance of 10^5^ cells ml^−1^ and mixed together in artificial communities (see below). These communities (40 μl) were used to inoculate 840 μl fresh beech-leaf tea (total volume 880 μl) in a deep well (1.2 ml 96-well microplates). The isolates selected were assembled randomly into *in vitro* microcosms with a bacterial inoculum of 500 cells total at each richness level.

### Experimental design

Artificial bacterial communities were set up using the random partitions design (Bell *et al.*, 2009). Briefly, the 16 isolates were assembled in communities (including monocultures) of manipulated richness levels 1, 2, 4, 8 and 16. Each of the isolates was represented once at each level of richness. Three partitions (combinations of microcosms, *n*=31) were established and independently replicated three times to give 93 experimental microcosms. Microcosms were incubated statically at 22 °C for a total of 7 weeks, with 300 μl of the culture removed and replaced by an equal volume of fresh beech-leaf tea every week to allow the bacterial stationary phase to be maintained along with the total volume of the microcosms postsampling. Samples (*n*=3) were collected on days 7, 28 and 49.

### Community productivity

After inoculation, the deep well plates were sealed using the MicroResp System (Macaulay Scientific Consulting Ltd, Aberdeen, UK) and the percentage of CO_2_ in the microcosm headspace was monitored in the connected indicator plates. These were made in accordance with the manufacturer's instructions. The absorbance (*λ*=572 nm) of the indicator plates were measured using a spectrophotometer (Synergy HT; BioTek, Swindon, UK) and subtracted from a starting absorbance value. The effect of atmospheric CO_2_ on the indicator plates was removed before analysis using a negative control. The MicroResp indicator (Macaulay Scientific Consulting Ltd) was calibrated to give a relationship (*R*^2^=0.936) between the mass (mg) of CO_2_ present and the change in absorbance: mgCO_2_ = e^(ln(Δλ_572_)+0.305414)/0.282164^, where Δ*λ*_572_ is the difference in absorbance (572 nm) between the beginning and ending of the experiment.

The abundance of bacterial cells was monitored using thiazole orange (Sigma-Aldrich) staining coupled with flow cytometry (C6 Accuri; BD Life Sciences, Oxford, UK). To a 25 μl sample of a microcosm, 75 μl of filtered Milli-Q water (Millipore, Watford, UK) with thiazole orange, to a final concentration of 420 nm. The samples were left to incubate with the stain for 5 min in the dark at room temperature. Flow cytometry analysed 10 μl of the stained sample, and appropriate gating (SSC/FL1 535/20 nm, particles smaller than 8000 FSC-H were excluded) was designed to exclude non-fluorescent debris from the media. As such, any particle that had fluorescence above 800 U was deemed to be a stained bacterial cell. Checks on selected microcosms indicated that cytometry yielded counts that were comparable to plate counts.

### Enzyme assays

The degree to which each community used one of three niche pathways to degrade biological matter present in the leaf-detritus medium, therefore obtaining nutrients (Sinsabaugh *et al.*, 1991; DeAngelis *et al.*, 2013), was assayed using substrates (Sigma-Aldrich) labelled with the fluorescent moiety 4-methylumbelliferone (MUB; Supplementary Table S2). Each of the substrates was incubated with a 25 μl sample of the microcosm to a final concentration of 40 μm (Frossard *et al.*, 2012) in filter sterilised water. Enzymes present in the sample cleaved the fluorescent moiety from the respective substrate backbone during 1-h incubation in the dark at room temperature. After this time, 10 μl of 1 m sodium hydroxide was added and the fluorescence (Ex/Em: 365 nm/445 nm) of the samples was measured immediately (Synergy HT; BioTek) for a period of 4 min and the maximum value recorded. Fluorescence values were converted to nanomolar MUB after normalisation across the data set; each value was multiplied by the fluorescence mean for the specific enzyme divided by the grand mean of fluorescence. To assess the degree of phenotypic plasticity or evolution within the microcosms samples grown to each of the different timepoints, which had been stored in freezing solution (30% (v v^−1^) glycerol with 0.85% (w v^−1^) NaCl) at −80 °C, were revived in fresh beech-leaf tea media and grown for a further 7 days at 22 °C. After this incubation, the extent to which the microcosms could degrade the substrate were repeated as described above.

### Molecular analysis

The composition of each microcosm community, and relative abundance of the isolates, was determined using terminal restriction fragment length polymorphism profiling at each timepoint. DNA was extracted using the ZR Fungal/Bacterial DNA Kit (Zymo Research, Irvine, CA, USA) following the manufacturer's protocol. A 1465 bp fragment of the 16S rRNA gene was amplified from all the microcosms using RedTaq Ready Mix (Sigma-Aldrich) and the primer set 27f/1492r (Sigma-Aldrich) for sequencing by Macrogen (Amsterdam, Netherlands). PCR cycling parameters were as previously described in Griffiths *et al.* (2011): initial denaturation at 95 °C for 5 min, followed by 30 cycles of 30 s at 95 °C, 56 °C and 72 °C, with a final extension time of 7 min at 72 °C. In a separate reaction, an 880bp fragment was amplified using the same reaction conditions using the primer set 27f-6FAM/ 907r. Amplified 16S rRNA gene regions were cleaved using a cocktail of the restriction endonucleases *Hha*I and *Rsa*I (Fisher Scientific, Loughborough, UK) and assessment of terminal restriction fragments were as described previously (Griffiths *et al.*, 2011).

### Statistical analysis

The predicted functioning was calculated assuming that all isolates were equivalent in abundance within the microcosm and assigned that proportion of their productivity in monoculture; 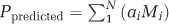, where *N* is the number of isolates in the microcosm, *a*_*i*_ is the abundance of the *i*th isolate in the microcosm if all isolates were equivalent and *M*_*i*_ is the functioning observed for the *i*th isolate in monoculture. We compared this approach to the one used by Foster and Bell (2012), which did not scale the predicted functioning by the predicted mixture relative abundances: 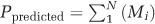. The contribution of each isolate within a mixture was derived by multiplying the observed total microcosm functioning by the relative abundance (proportion) of each isolate within the assemblage (measured at the same timepoint); *P*_*ij*_=*ra*_*ij*_*P*_observed,*j*_, where *P*_*ij*_ is the functioning of the *i*th isolate in the *j*th microcosm, *ra*_*ij*_ is the relative abundance of the *i*th isolate in the *j*th microcosm and *P*_observed,*j*_ is the observed overall functioning of the *j*th microcosm.

If bacteria could not be differentiated by their terminal restriction fragment, the relative abundance for a non-unique terminal restriction fragment was shared equally (the null hypothesis) by the number of isolates that associated with that fragment. We have investigated whether our results are sensitive to this assumption by taking the most extreme alternative scenario: rather than distributing the abundance values evenly among the isolates, we assumed that all individuals belonged to one of the isolates (chosen at random). We found that the results were not sensitive to analysing the results in this way (compare Figure 3 with Supplementary Figure S2).

Observed and predicted levels of functioning were used to calculate a response ratio (Goldberg *et al.*, 1999) to estimate the role of interspecific interactions in altering isolate productivity: RR=*P*_observed_/*P*_predicted_. These ratios were log_e_ transformed and represent the impact of a given population on the functioning attributed to a particular isolate.

In addition, we estimated the importance of complementarity and selection using the additive partitioning equation (Loreau and Hector, 2001): 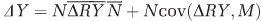, where Δ*Y* is the difference between the observed and expected per-capita respiration of mixtures, *N* is the number of species in the mixture, Δ*RY* is the difference between the per-capita respiration of the proportion seeded of a particular isolate in monoculture and the per-capita respiration of the same isolate while in the mixture and *M* is the monoculture functioning. We obtained per-capita respiration rates in the mixture by multiplying the relative abundance of each species in the mixture by the overall respiration of the mixture. In the equation 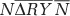 (‘complementarity effect') reflects the degree to which respiration depends on niche differences, whereas *N*cov (Δ*RY, M*) (‘selection effects') reflects the importance of a particular species in setting the level of respiration.

All analyses on this data set were performed using linear models after the assumptions for using parametric statistics had been confirmed visually. If the data violated the assumptions, then the data were log_e_ transformed. All corrections on multiple comparisons (denoted by a *P*_adj_ value) were Bonferroni, unless stated in the text, and for all statistics *α*=0.05. For the repeated-measures analysis of variance normality and homogeneity were improved by log_10_ transformation. Statistical interactions between ‘microcosm' and ‘time' and between ‘microcosm' and ‘richness' were checked. According to that, additive or non-additive models were applied in the Error structure. The ‘biology' package (Logan, 2010) was used to calculate sphericity with a type II model was used to correct for the unbalanced richness levels. All statistical analyses and visualisations were performed in R (v.2.15.2 and v.3.2.0) statistical environment.

## Results

### Community and ecosystem dynamics

**Figure 1:**
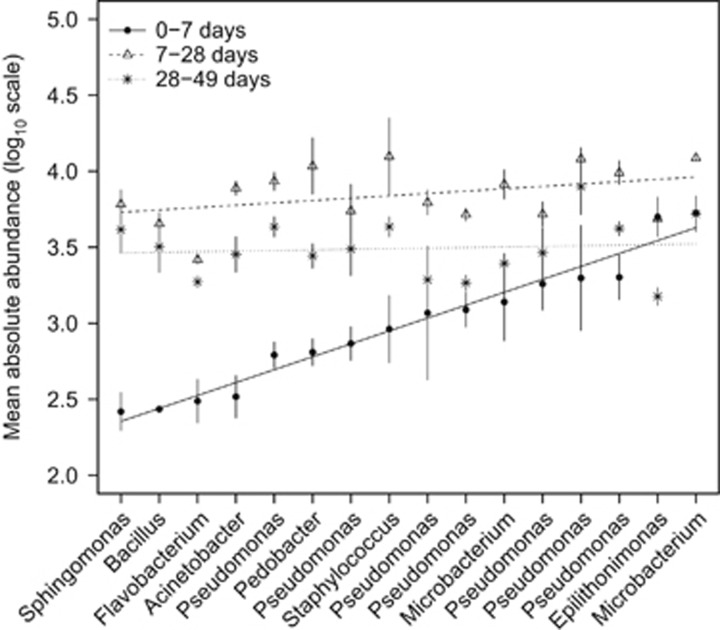
The mean abundance (cell counts ml^−1^) of each of the isolates counted using the total microcosm cell counts multiplied by the relative terminal restriction fragment length polymorphism (tRFLP) band intensities of each isolate within a given microcosm. The isolates were ranked based on their abundance after 7 days coculture. These data show that those isolates that are rarest at 7 days increase to the same level as the dominant isolates by the conclusion of the experiment. Regression lines and means±1 s.e. are presented.

We tracked the abundance of each of the 16 isolates in each microcosm at 7, 28 and 49 days postinoculation. We calculated absolute counts of each isolate averaged across the experiment, and ranked the abundances (Figure 1; abundance changes in individual isolates is shown in Supplementary Figure S3). The data indicated that there were initially (day 7) strong differences in overall ranked abundance (slope at day 7 (*β*_day7_)=0.08, F_1,14_=359.9, *P*<0.001; Figure 1), but this variation in abundance was not maintained. Isolate abundances had equilibrated at subsequent timepoints, with reductions in magnitude and significance across the range of isolate abundances (*β*_day28_=0.02, F_1,14_=2.5, *P*=0.14; *β*_day49_=0.01, F_1,14_=1.4, *P*=0.72; Figure 1).

**Figure 2:**
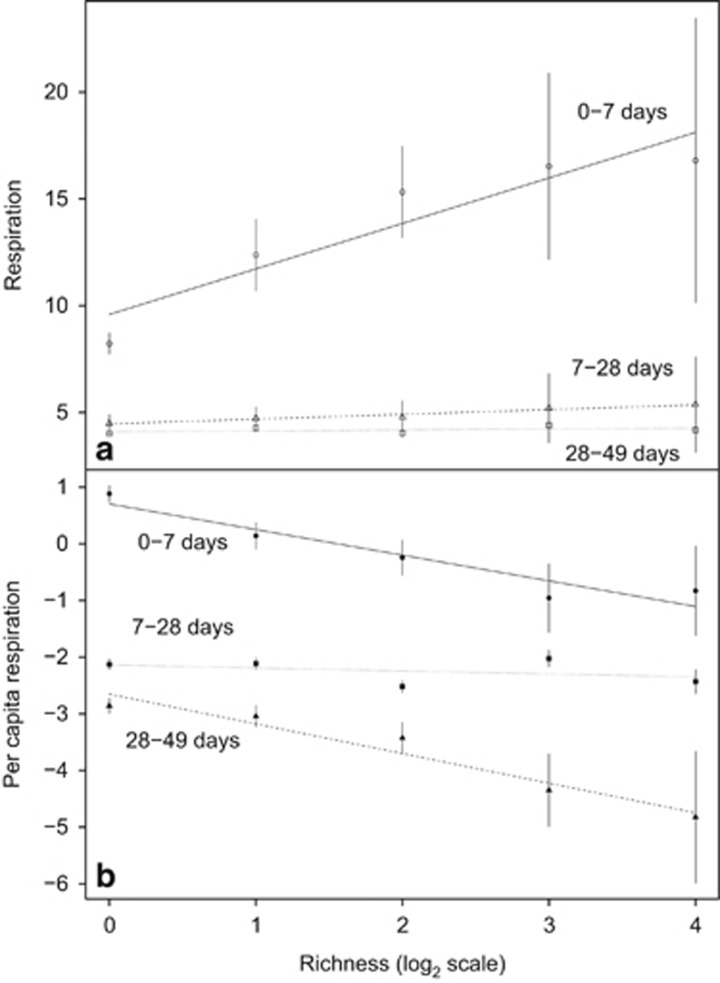
The effect of increased richness on bacterial respiration rate. The respiration rate per day (μg CO_2_ per day) of the microcosms were found to increase with increasing richness (**a**). The log_e_-transformed per-capita respiration rate (pg CO_2_ per day per isolate) was found to decrease with increasing diversity of the microcosms (**b**). Both sets of data illustrated that over the three timepoints sampled, the relationship became shallower until, after 7 weeks, where only a slight negative trend was observed. Regression lines and means±1 s.e. are presented.

We also tracked changes to community respiration and community abundance (total cell counts). The results showed that the mean (±1 s.e. throughout) quantity of CO_2_ produced after 7 days (11.0±0.7 μg CO_2_ per day) was higher compared with that at either 28 (4.7±0.3 μg CO_2_ per day) or 49 days (4.1±0.1 μg CO_2_ per day), with increasing diversity causing an overall increase in respiration at all timepoints (Figure 2a). In contrast, the mean number of cells per microcosm was highest at 28 days (13.0 × 10^4^±1.3 × 10^4^ cells ml^−1^), an increase from 7 days (28.9 × 10^3^±4.7 × 10^3^ cells ml^−1^) and higher compared with those detected at 49 days (56.9 × 10^3^±5.3 × 10^3^ cells ml^−1^). We calculated per-capita respiration rates by dividing the respiration rate by the number of cells (Figure 2b). These data showed that per-capita respiration rate decreased over time with the highest mean rate recorded after 7 days (5.3±0.6 pg CO_2_ per cell per day), which decreased after 28 days (0.22±0.05 pg CO_2_ per cell per day) and was slowest at 49 days (0.18±0.01 pg CO_2_ per cell per day). The relationship between per-capita respiration rate and richness changed significantly over time (repeated-measures analysis of variance, F_4,274_=8.1, *P*<0.001) with a negative slope between bacterial richness and per-capita respiration at the first sampling point (*β*_day7_=−0.43 μg CO_2_ per cell per isolate per day, F_1,277_=5.1, *P*=0.03). The slope declined at 28 days (*β*_day28_=−0.02 pg CO_2_ per cell per isolate per day, F_1,277_=1.5, *P*=0.23) and further at the conclusion of the experiment at 49 days (*β*_day49_=−0.01 pg CO_2_ per cell per isolate per day, F_1,277_=4.8, *P*=0.03).

### Species interactions during succession

**Figure 3:**
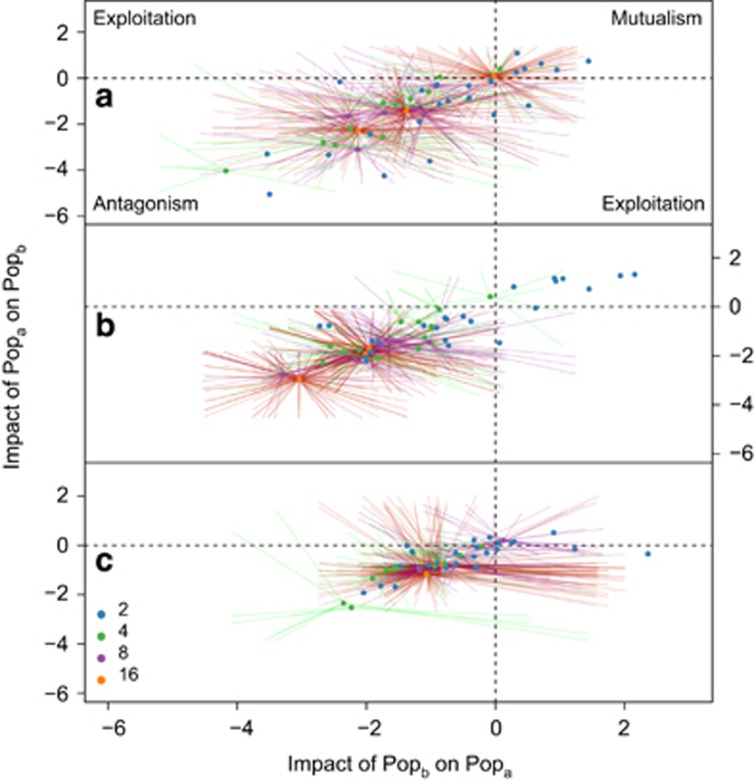
The pairwise impact of each isolate population on co-occurring isolates. Interactions were calculated using the difference between the predicted per-capita respiration and that which was observed at the individual isolate population level at (**a**) 7, (**b**) 28 and (**c**) 49 days. Solid points represent the mean interaction within a microcosm. The terminus of each line is the interaction between each pair of species within the microcosm. Axis scales represent the log-transformed response ratio of the per-capita respiration rates.

We inferred pairwise interactions between isolates in mixtures by comparing the individual contribution to the microcosm functioning of each isolate in mixture to their equivalent productivity in monoculture. These values, incorporating changes in both per-capita respiration and relative abundance, were used to create a relative per-capita productivity ratio for every pairwise interaction included in the experiment. For each isolate pair, reductions in the productivity of both species relative to their monoculture value (−/− interaction) indicated antagonism, increases in the productivity of both species (+/+) indicated mutualism, whereas increases in one and reductions in the other (+/−) implied exploitation. We observed three strong patterns. First, interactions were primarily antagonistic (−/−) interactions (Figure 3), with a slight increase observed with increasing richness (Supplementary Figure S4). Second, the variance (5.12, 6.18 and 1.48 for the interactions at times 7, 28 and 49 days, respectively, Fligner–Killeen test *χ*^2^_2_=73.3, *P*=0.001) and mean interaction (F_2,267_=5.51, *P*=0.005; all pairwise *t*-tests between days *P*_adj_<0.001 following Bonferroni correction) attenuated significantly at the conclusion of the experiment. After 7 days of coculture, the mean log-transformed interaction was −1.02 (±0.14). There was subsequently a significant increase in the mean interaction to −3.33 (±0.14) at day 28 and then a significant decrease to −0.32 (±0.07) at day 49. Third, interactions were symmetric, for a pair of species, the impact of the first on the second was closely related to the effect of the second on the first (Figure 3). For comparison with previous work (Foster and Bell, 2012), we found a qualitatively similar trend when the predicted mixture functioning was calculated simply as the sum of the constituent monoculture functioning (Supplementary Figure S5).

The effect of richness on the mean interaction strength showed that at all of the timepoints there was an increasing relationship (Supplementary Figure S4); at the highest diversity levels, the mean interaction strength was higher compared with that at the lower levels of richness. At the first timepoint, a nonsignificant relationship was observed (*β*_day7_=0.06, F_1,118_=0.9, *P*=0.35). This trend continued for the remainder of the experiment (*β*_day28_=0.04, F_1,118_=0.3, *P*=0.60; *β*_day49_=0.01, F_1,118_=0.03, *P*=0.85).

**Figure 4:**
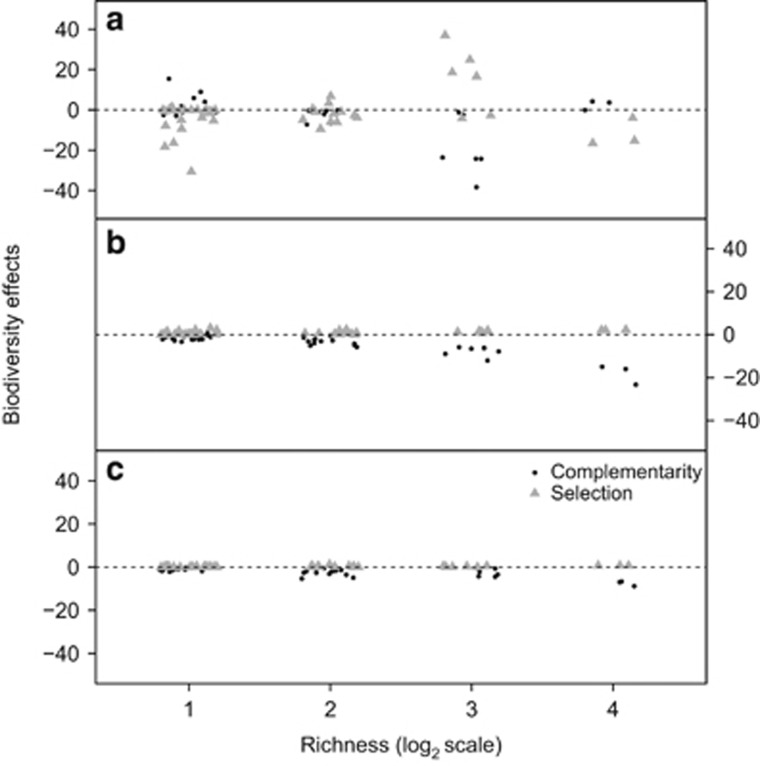
The role of biodiversity effects on the microcosms during succession. Higher order biodiversity effects were categorised into selection and complementarity effects using the additive partitions design. At the start of the experiment, (**a**) both selection and complementarity have varied contributions at each richness level; however, as the length of the experiment increases, the contributions of the selection decreases and the complementarity has a greater effect and definite trend at the mid-point (**b**), whereas the microcosms decrease in interactions at the end of the experiment (**c**).

We partitioned the effect of diversity on per-capita respiration into complementarity and selection using the additive partitioning equation after (Loreau and Hector, 2001) (Figure 4). The degree of complementarity in the microcosms declined with increasing richness at the outset of the experiment (*β*_day7_=−5.07, F_1,43_=6.15, *P*=0.017). The influence of complementarity changes over subsequent sampling periods (*β*_day28_=−4.53, *β*_day49_=−1.64 pg CO_2_ per cell per day per isolate, F_1,43_<60.40, *P*<0.001). In contrast, selection was positively associated with increasing richness at the outset of the experiment (*β*_day7_=5.08, F_1,43_=2.05, *P*=0.160). The selection effect dissipated over the course of the experiment (*β*_day28_=0.44, *β*_day49_=0.16) but became more dependent on richness (F_1,43_<13.81, *P*<0.001). In summary, there was a decline in species complementarity mirroring the decline in per-capita respiration (Figure 2b) and the reduction in the strength of pairwise interactions (Figure 3).

### Substrate degradation during succession

**Figure 5:**
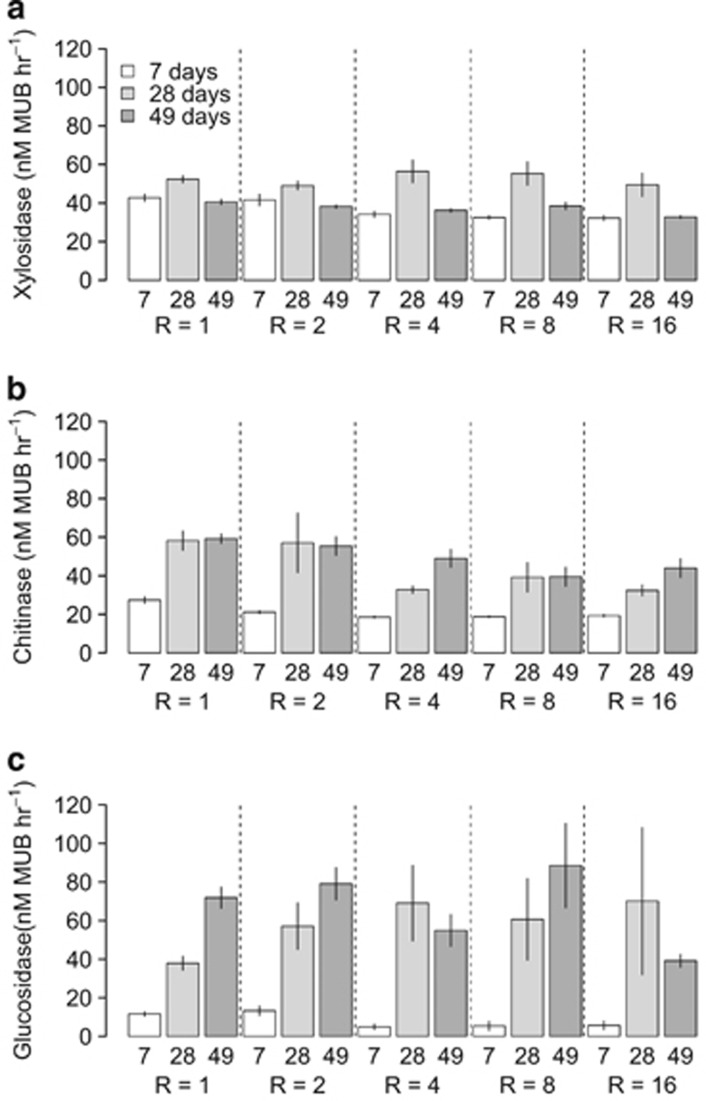
Effect of microcosm richness and time on the concentration of MUB (nm) cleaved from the specific substrate. Mean concentrations (vertical lines represent ±1 s.e.) of the fluorescent moiety MUB released from one of three, progressively more recalcitrant substrates by its appropriate enzyme: hemicellulose by xylosidase (**a**), chitin by chitinase (**b**) and cellulose by β1,4-glucosidase (**c**). Microcosms were incubated for 7 (white), 28 (light grey) and 49 (dark grey) days. Timepoints are grouped based on the richness level (*R*) of the microcosms.

We measured substrate degradation in each microcosm by measuring the ability of the community to cleave the fluorescent moiety (MUB) from three different substrates (Figure 5). Xylosidase metabolises the labile substrate xylose—a monomer prevalent in hemicellulose—whereas β-chitinase and β-glucosidase break down chitin—microbial cell wall component—and cellulose, respectively. There were significant differences in substrate degradation rates across the three substrates after 7 days, across all richness levels (pairwise *t*-tests *P*_adj_<0.001), with the xylosidase enzyme the most active (40.4±1.3 nm MUB h^−1^) compared with chitinase (23.8±1.0 nm MUB h^−1^) and β-glucosidase (10.5±0.9 nm MUB h^−1^), which is shown to be less active. This trend shifted over time, with no significant differences between the activities of the three enzymes after 28 days (mean activity for the β-glucosidase, chitinase and xylosidase at 49.4±4.8, 52.6±4.8 and 52.1±1.4 nm MUB h^−1^, respectively). The trend had reversed after 49 days, with β-glucosidase now significantly (pairwise *t*-tests; *P*_adj_<0.001) the most active (71.6±4.0 nm MUB h^−1^) with chitinase (55.1±1.9 nm MUB h^−1^) and xylosidase (39.0±0.8 nm MUB h^−1^) less active. This trend shift over time was conserved when increasing richness was included in a repeated-measures analysis of variance. The results indicated that for the activity of xylosidase, there were no significant effects of either richness (F_4,274_=1.4, *P*=0.25) or time (F_1,274_=0.0, *P*=0.95). In contrast, chitinase activity was not affected by bacterial richness (F_4,274_=1.3, *P*=0.27), but there was a significant effect of time (F_1,274_=327.1, *P*<0.001), with the highest activity at the end of the experiment. In contrast, the activity of β-glucosidase was found to have a significant relationship with both richness (F_4,274_=4.0, *P*=0.004) and time (F_1,274_=419.3, *P*<0.001). There was also a significant interaction between richness and time (F_4,274_=4.0, *P*=0.003).

The changes in substrate use might be because of some degree of plastic metabolic responses, evolutionary changes, changes in abundance (ecological sorting), changes in substrate availability over time or a combination. To control for the effect of substrate availability, we grew each of the communities from every timepoint in a common environment (fresh media) and measured substrate degradation (Supplementary Figure S6). We predicted that substrate degradation would be constant across timepoints if the response was because of metabolic plasticity or species sorting, as both would be able to respond rapidly to the new environment independent of which timepoint they were taken from. Conversely, an evolutionary response would be implied if communities collected from different timepoints had different substrate degradation rates when collected from different timepoints. Repeated measures analysis of these data found that there was a marginally significant relationship between the activity of xylosidase and time (*β*=0.004, F_1,834_=3.9, *P*=0.05) but not between activity and richness (F_1,834_=1.0, *P*=0.33). In contrast, both other enzymes had significant positive relationships with time (*β*_chitin_=0.07, *β*_glucosidase_=0.15, F_1,834_>102.9, *P*<0.001) and richness (*β*_chitin_=0.01, *β*_glucosidase_=0.01, F_1,834_>10.2, *P*<0.002). These trends were stronger in low richness communities. Altogether, this indicated that the significant change in enzyme activity per cell for labile carbon were at best weakly related to genetic change (and therefore likely reflect phenotypic plasticity), whereas changes to the activity of the more recalcitrant carbon were consistent with evolutionary change of the isolates.

## Discussion

There have been many studies of microbial community dynamics, but most have described compositional changes without quantifying how biotic interactions change over time. We show a clear decline in the strength of interspecific interactions over time. This result is consistent with a shifting pattern of substrate usage over the course of the experiment. The enzyme assays demonstrated a shift away from labile polysaccharides (hemicellulose) to long-chain, fibrous cellulose. Evidence for this shift was observed using the activity of chitinase and β-glucosidase. Chitinase activity increases at 28 days and remained constant for the rest of the experiment, whereas β-glucosidase showed a steady increase in activity throughout the experiment and peaked at 49 days. The results are consistent with our *a priori* classification of the substrates (labile to recalcitrant), with recalcitrant cellulose requiring considerably more energy to release the carbon (Gupta *et al.*, 2012), and was therefore used only when other carbon sources were exhausted or at low concentration. One mechanism by which chitin is degraded is by deamination to cellulose (Beier and Bertilsson, 2013); thus, the observed increase in the β-glucosidase is consistent with a proposed shift in resource use of the bacteria within the microcosms. Further evidence to support this conclusion comes from the growth data (Figure 1), which show a decrease in bacterial growth, presumably because of changes in resource availability (Roller and Schmidt, 2015), mirroring the shift to using more recalcitrant carbon sources.

We speculate that this shift in substrate usage is the mechanism underlying the shift in interactions over time. While the metabolic pathways necessary to break down labile substrates are expected to be widely available across the strains used in the study, breakdown of recalcitrant substrates requires more specialised metabolic pathways, potentially selecting for specialist species though further work would be needed to confirm this conclusion. Indeed, the activity of the xylosidase in monoculture isolates was more consistent than either the chitinase or the β-glucosidase (Supplementary Figure S7). It is this shift from labile carbon use in early succession to more recalcitrant in late succession that might result in the observed shift toward more neutral interactions over time, as these carbon sources are more difficult to breakdown and can give greater opportunity for niche complementarity. Eisenhauer *et al.* (2013) reported that in highly nutrient complex environments, niche complementarity becomes increasingly important while selection of specific bacteria becomes less important. Our results build on this result in finding that it is not the complexity of the environments alone that determines the relative role of synergistic interactions as we would not expect complementary interactions to be important in complex environments, consisting of labile substrates. As the nutrients available become more recalcitrant, the opportunities for the isolates to specialise increases (Crespi, 2001). Further work, for example, to assess gene expression the metabolome, might be able to confirm this switch in metabolic activity.

We observed evidence that suggested that at higher levels of diversity became more complementary over time (Figure 4), although we did not detect a relationship between diversity and the strength of the pairwise interactions (Supplementary Figure S4). We speculate that this pattern could have resulted from the diversification of isolates into distinct or complementary niches, with some bacteria potentially able to use secondary metabolites that are unused by other species (Lawrence *et al.*, 2012). Alternatively, the pattern is consistent with the accumulation and sharing of costly periplasmic enzymes to degrade recalcitrant molecules (Cunha *et al.*, 2010) in environments where space is at a premium. Finally, a third possibility is that, in more diverse communities, those species in direct competition at lower diversities could be suppressed by third-party interactions, with the additional interactions cancelling each other out. Distinguishing among these mechanisms could provide an interesting avenue for future experiments.

We demonstrate a substantial reduction in the strength of negative interactions during the succession of these communities (Figure 3 and Supplementary Figure S2). The result is consistent with the idea that strong interactions destabilise communities through a variety of ecological mechanisms (for example, by increasing the likelihood of extinction), thus weak interactions tend to be observed in real communities (McCann *et al.*, 1998; Kokkoris *et al.*, 1999; Coyte *et al.*, 2015). This result is among the first to show how observed interactions reflect the ‘ghost of competition past' (Connell, 1980), but this dynamic had not previously been recorded during bacterial succession.

The diversity level in this study is based on isolates that were chosen based on their ability to use nutrient sources, by way of enzyme production, before the start of the experiment. As such we have included a number from the genera *Pseudomonas*. These are one of the most common bacteria in our study ecosystem (beech treeholes), and are also one of the most diverse in terms of ecological space (as reviewed in Silby *et al.*, 2011). In this study, we observe a small part of this diversity as each of the isolates behaves differently in the microcosms (Supplementary Figures S3 and S7). We explored whether communities that were dominated by pseudomonads were unusual (for example, displayed stronger negative interactions, or lower-than-expected functioning), but could find little evidence for such an effect.

Usage of labile substrate did not differ among communities collected from different timepoints and different levels of diversity. In contrast, usage of more recalcitrant substrates was clearly affected by the treatments, even when the communities were placed in a common environment. The results demonstrate that the usage of labile substrates in the experiment is likely due to either metabolic plasticity or to rapid species sorting. Although this observed change could have been due to the extinction of particular isolates, causing the loss of some functionality, the terminal restriction fragment length polymorphism results suggested that none of the species dropped below the level of detection for the analysis. Differential usage of recalcitrant substrates in the experiment, however, appears to be due to evolutionary or physiological changes that are not easily reversed. We believe that the relative importance of ecological versus evolutionary processes in complex microbial communities remains understudied (Barraclough, 2015), and understanding the dynamics of the resource environment might provide valuable insight into their relative roles in nature.

In conclusion, we found that over the course of the experiment there was a reduction in bacterial interactions, which was accompanied by an apparent shift in resource utilisation. We think this study highlights an important mechanism in bacterial ecology, which could be tested through transcriptomic/metabolomic approaches. These results support previous modelling reports that indicate a reduction of strong interactions over time, as well as suggesting a mechanism for these phenomena.
