# Supplementary material for: Resource-dependent attenuation of species interactions during bacterial succession
Source: ISME J. 2016 Feb 19;10(9):2259–68. doi: 10.1038/ismej.2016.11 (PMC4989303; doi:10.1038/ismej.2016.11)
Supplement: Supplementary Table S1 [file ismej201611x1.docx]

Figure S1. Hierarchical clustering of the isolates (a) based on their substrate utilisation profiles (measured by fluorescence cleaved from a specific substrate). Principle Component Analysis based on the ability of the isolates to cleave fluorescence from a specific substrate (b). PC1 is the ability to cleave recalcitrant resources (cellulose), PC2 is the ability to cleave simpler resources (hemi-cellulose & chitin).

Figure S2. The pairwise interactions as calculated for Figure 3, but in communities where species share a TRF, we allocated all of the functioning to one of the species (chosen at random) rather than dividing the functioning evenly between the isolates. It is clear that the patter is virtually unaltered compared to Figure 3, with the same pronounced diminution in interactions during the final time period (bottom panel).

Figure S3. Mean abundances of each isolate at each timepoint at a given richness level. Axis labels: x-axis represents time (at 7, 28 and 49 days) with the y-axis presented are the same at intervals of 1 log_10_. Dotted line represents the mean trend of the isolate across the course of the experiment. Dots are jittered around each time point for visualisation

Figure S4. The mean pairwise interaction strength at the different richness levels at each timpoint. The data indicates that there was a rising trend for interactions to be less antagonisitic at the higher levels of diversity across the experiment.

Figure S5. The pairwise impact of each isolate population on co-occuring isolates. Interactions were calculated using the difference between the predicted per capita respiration based upon the additive assumption (Foster & Bell, 2012) and that which was observed at the individual isolate population level. Axes scales represent the log tranformed response ratio of the per capita respiration rates.

Figure S6. The difference between the concentration of MUB cleaved from specific substrates by bacterial communities that had been grown for 7, 28 and 49 days in BLT. Mean concentration of the fluorescent moiety MUB released from one of three, progressively more recalcitrant substrates by its appropriate enzyme; hemi-cellulose by xylosidase (a), chitin by chitinase (b) and cellulose by β1,4 glucosidase (c). Microcosms were incubated for 7 (white), 28 (light grey) and 49 days (dark grey). Time points are grouped based on the richness level (R) of the microcosms. Error bars represent ±1 standard error of the mean. Performances of populations from day 7 can be regarded as “ancestral”, while performance from populations from day 28 and day 29 can be regarded as “evolved”.

Figure S7. The degree of change for each of the isolates, in monoculture, for each of their enzyme activities at a given timepoint. Vertical lines represent one standard error.
