# Supplementary material for: Resource-dependent attenuation of species interactions during bacterial succession
Source: ISME J. 2016 Feb 19;10(9):2259–68. doi: 10.1038/ismej.2016.11 (PMC4989303; doi:10.1038/ismej.2016.11)
Supplement: Supplementary Table S2 [file ismej201611x2.docx]

Table S1. Molecular identification of bacterial isolates used in this study. Acc# represents the closest BLAST match on GenBank. tRF length is the length of the 5’-fragment of the 16S rRNA gene when amplified with the primer set 27f/1492r and digested with a *Hha*I-*Rsa*I endonuclease cocktail.

| **Isolate ID** | **Genus** | **Acc#** | **SeqID (%)** | **tRF (bp)** |
| --- | --- | --- | --- | --- |
| Cul_BB13_02_R | *Pseudomonas* | HQ224651 | 99.0 | 193 |
| Cul_BB13_03_R | *Pedobacter* | KC569794 | 96.0 | 89 |
| Cul_BB13_11_R | *Pseudomonas* | KC790251 | 100.0 | 202 |
| Cul_BB13_12_R | *Pseudomonas* | KC306440 | 100.0 | 202 |
| Cul_BB13_17_R | *Bacillus* | LN680100 | 100.0 | 485 |
| Cul_SP01_18_R | *Microbacterium* | LN615078 | 100.0 | 138 |
| Cul_SP01_19_R | *Pseudomonas* | HQ224588 | 99.9 | 202 |
| Cul_WYM24_20_R | *Pseudomonas* | KP216508 | 100.0 | 193 |
| Cul_WYM27_23_R | *Acinetobacter* | KC176452 | 100.0 | 87 |
| Cul_WYT16_30_R | *Pseudomonas* | HQ224617 | 99.9 | 193 |
| Cul_AE76_46_R | *Flavobacterium* | FJ889628 | 99.8 | 85 |
| Cul_AE79_49_R | *Sphingomonas* | KM891564 | 100.0 | 76 |
| Cul_AE80_53_R | *Staphylococcus* | KM378596 | 100.0 | 234 |
| Cul_AE83_55_R | *Pseudomonas* | JQ977348 | 99.6 | 193 |
| Cul_AE85_56_R | *Microbacterium* | KM507662 | 100.0 | 138 |
| Cul_AE90_59_R | *Epilithonimonas* | JX293123 | 99.2 | 88 |
