# Supplementary material for: Resource-dependent attenuation of species interactions during bacterial succession
Source: ISME J. 2016 Feb 19;10(9):2259–68. doi: 10.1038/ismej.2016.11 (PMC4989303; doi:10.1038/ismej.2016.11)
Supplement: Supplementary Figures [file ismej201611x3.docx]

Table S2. Substrate analogues used to assess extracellular enzyme activity.

| ***Substrate*** | ***Function*** | ***Enzyme*** | ***Nature of resource*** |
| --- | --- | --- | --- |
| MUB-β-D-xylopyranoside | Hemicellulose degradation | Xylosidase | Labile |
| MUB-*N*-acetyl-β-D-glucosamine | β-1,4-glucosamine degradation | Chitinase | Intermediate |
| MUB-β-D-glucopyranoside | Cellulose degradation | Β-glucosidase | Recalcitrant |
