# Supplementary figures and images for: Resource-dependent attenuation of species interactions during bacterial succession
Source: ISME J. 2016 Feb 19;10(9):2259–68. doi: 10.1038/ismej.2016.11 (PMC4989303; doi:10.1038/ismej.2016.11)

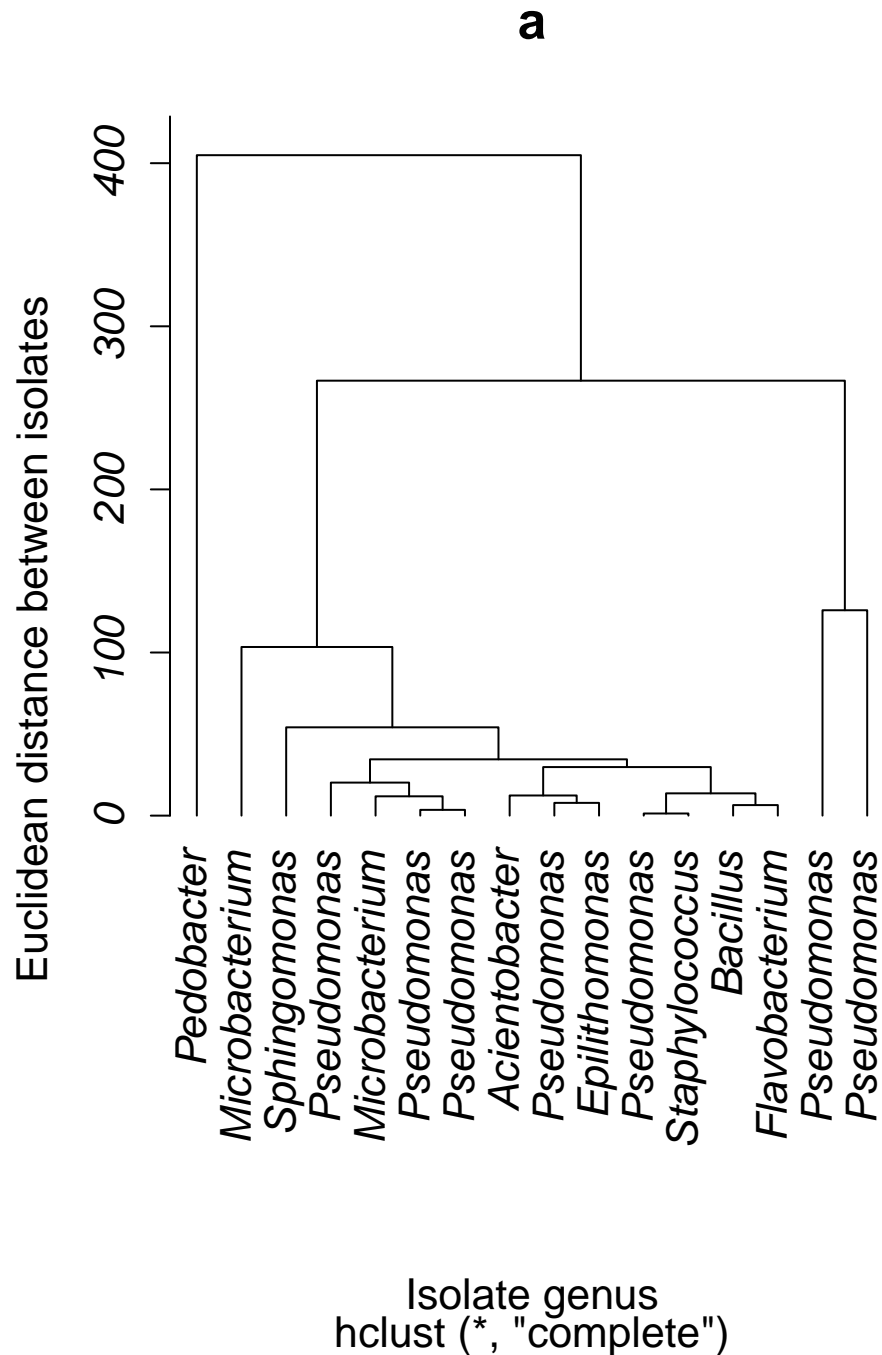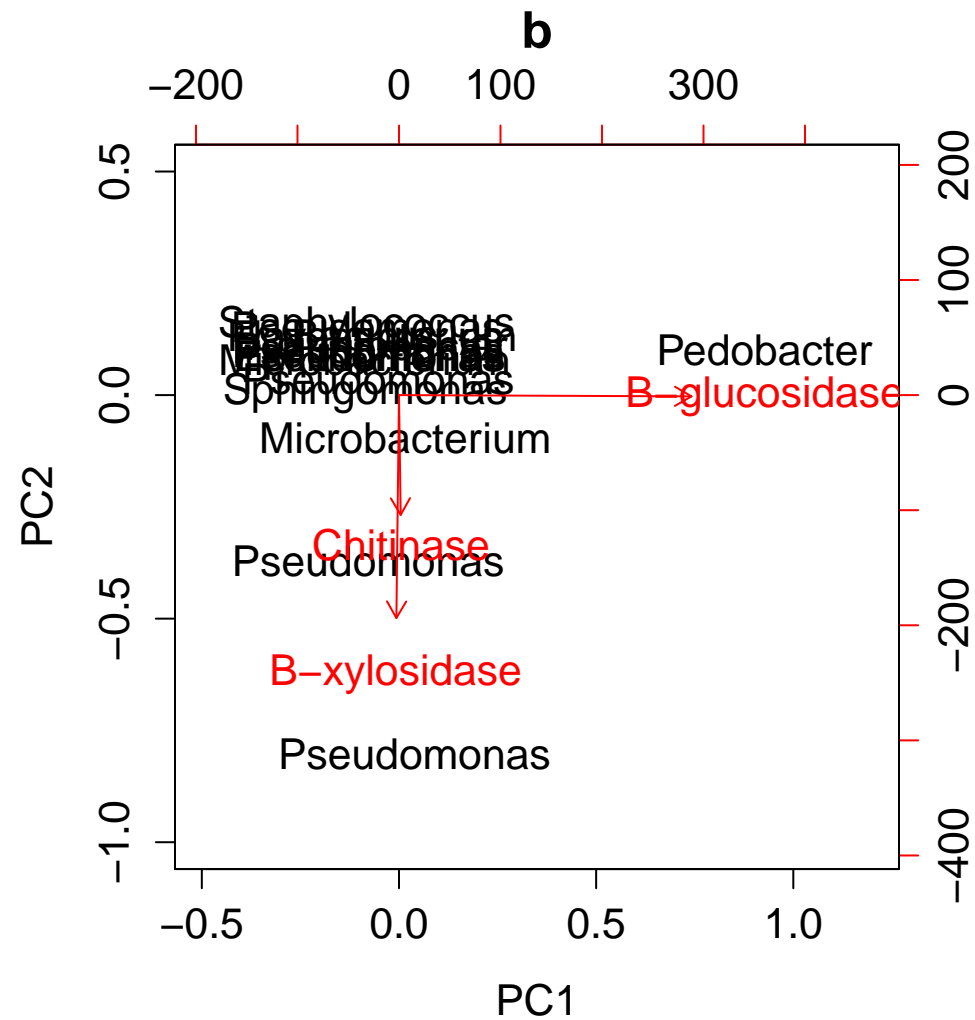

Supplement: Supplementary Figure S1 [file ismej201611x4.pdf]

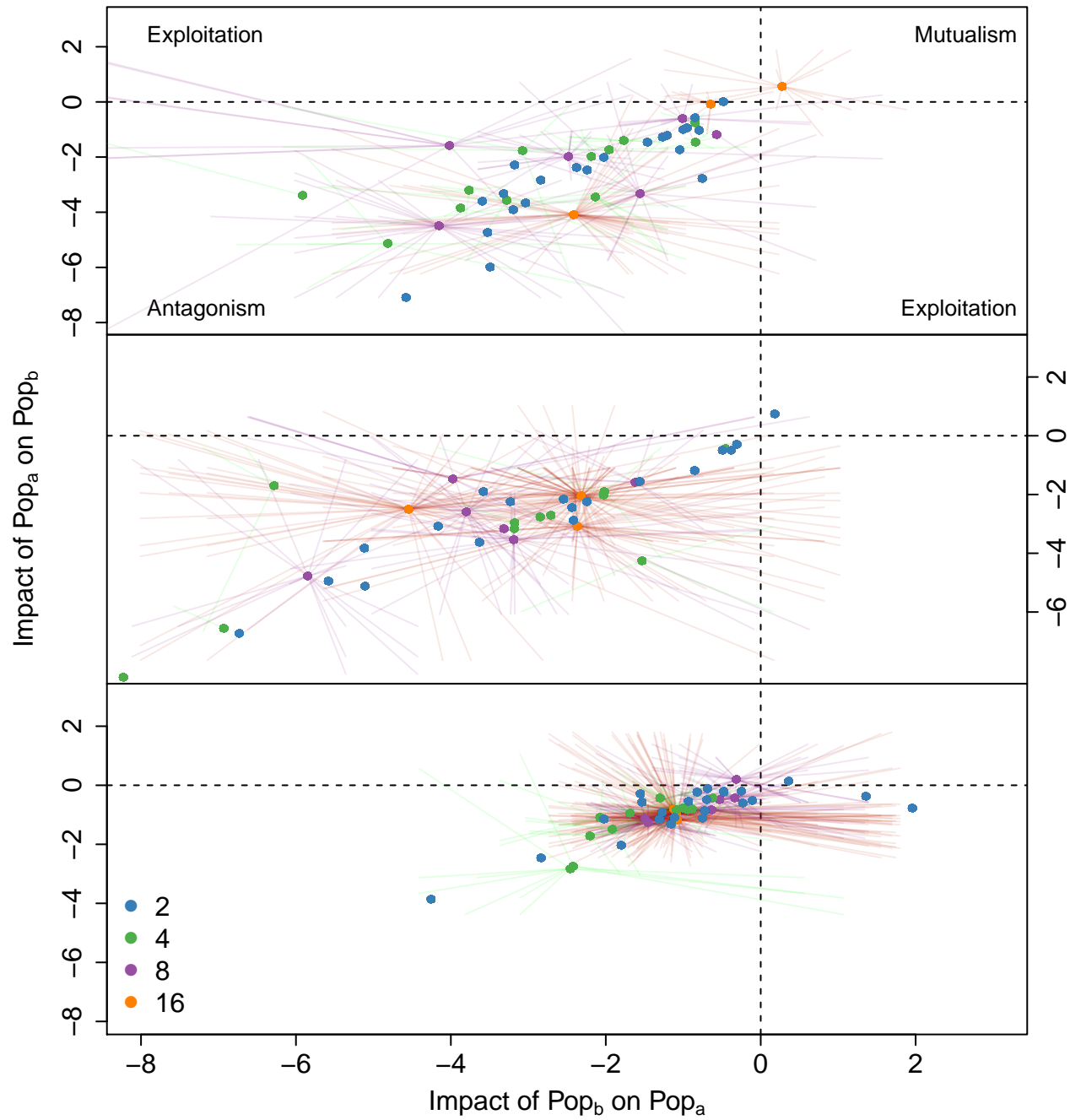

Supplement: Supplementary Figure S2 [file ismej201611x5.pdf]

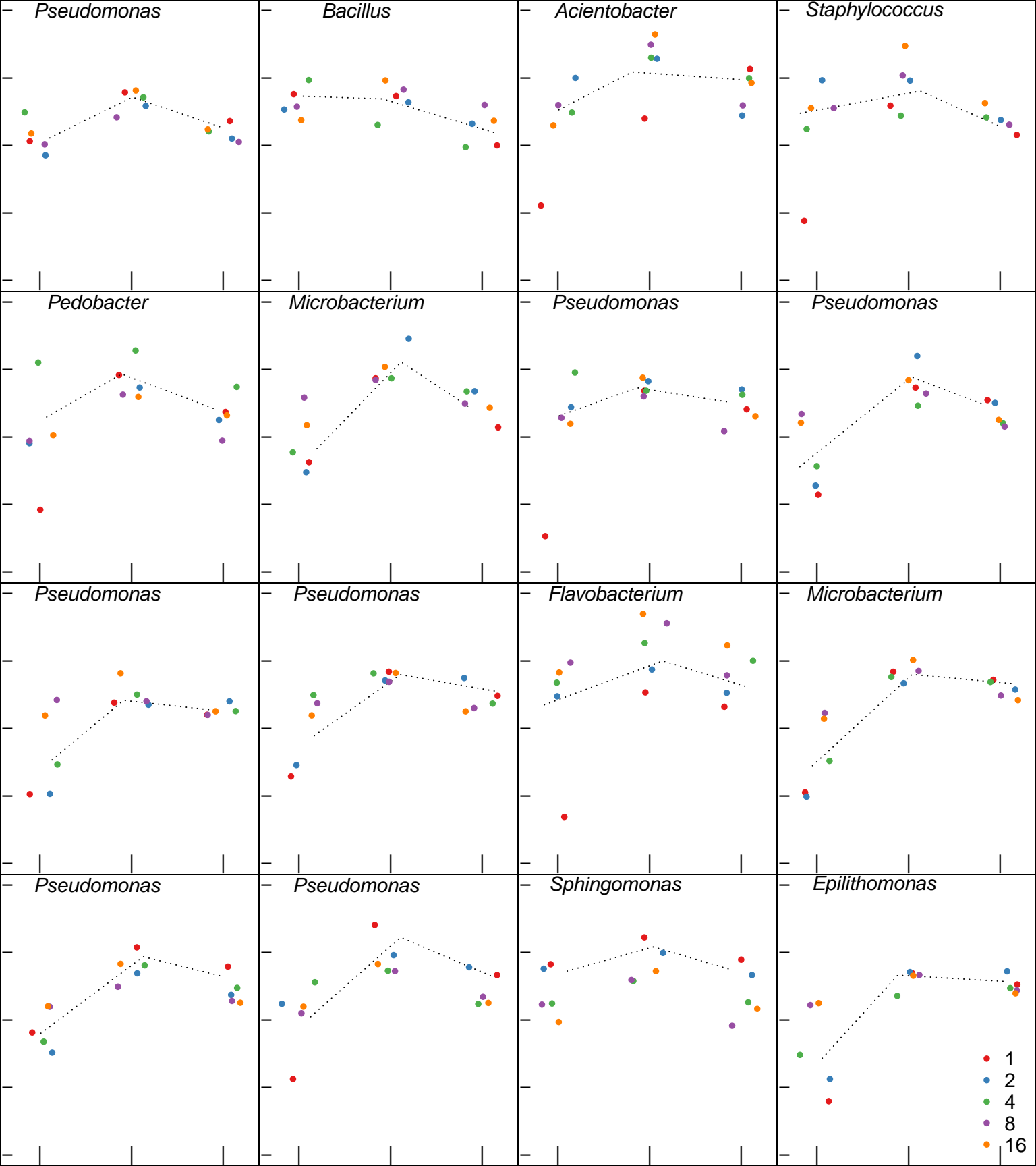

Supplement: Supplementary Figure S3 [file ismej201611x6.pdf]

Mean interaction strength

- 0–7 days
- △ 7–28 days
- \* 28–49 days

Richness ( $\log_2$  scale)

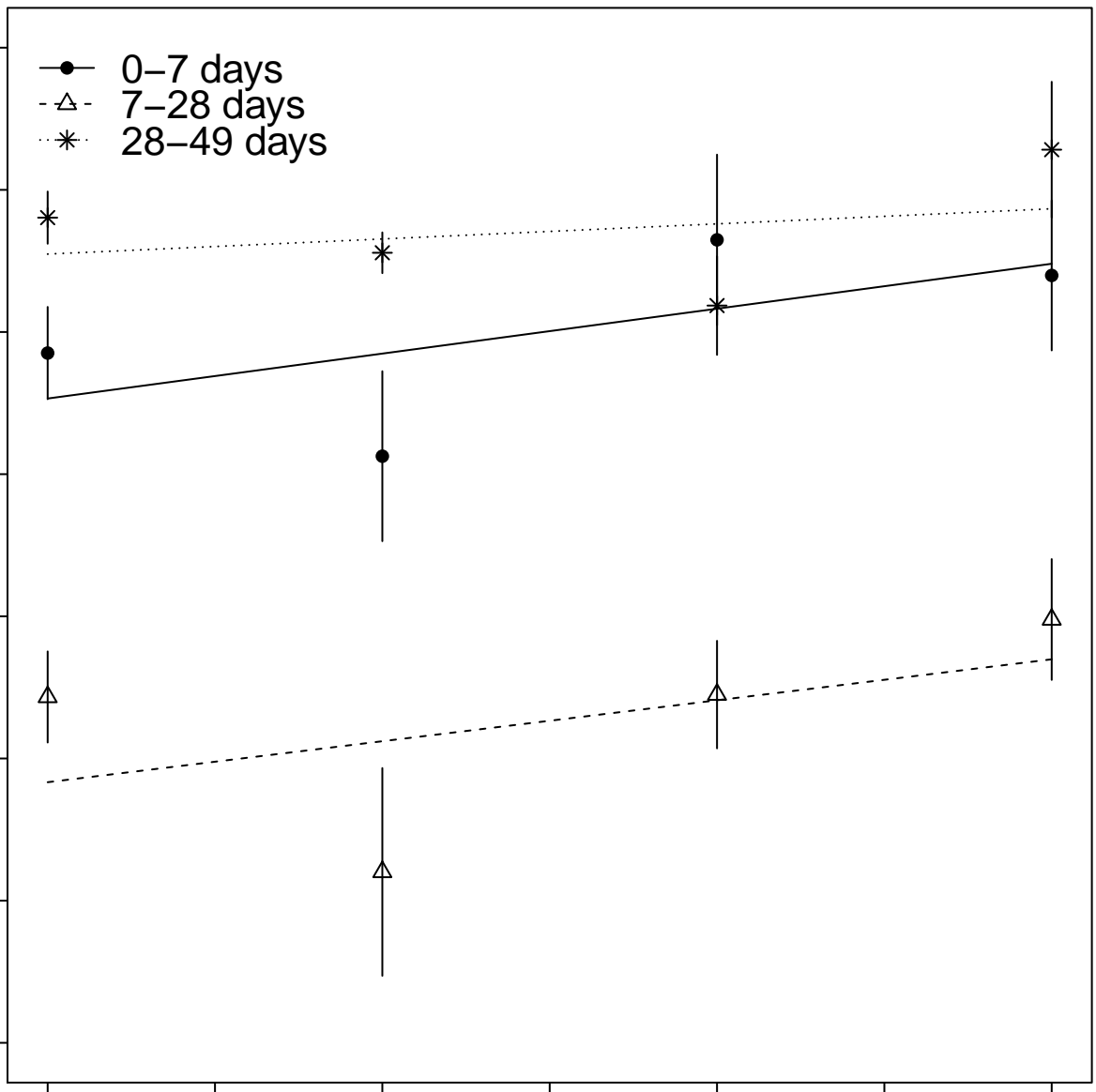

Supplement: Supplementary Figure S4 [file ismej201611x7.pdf]

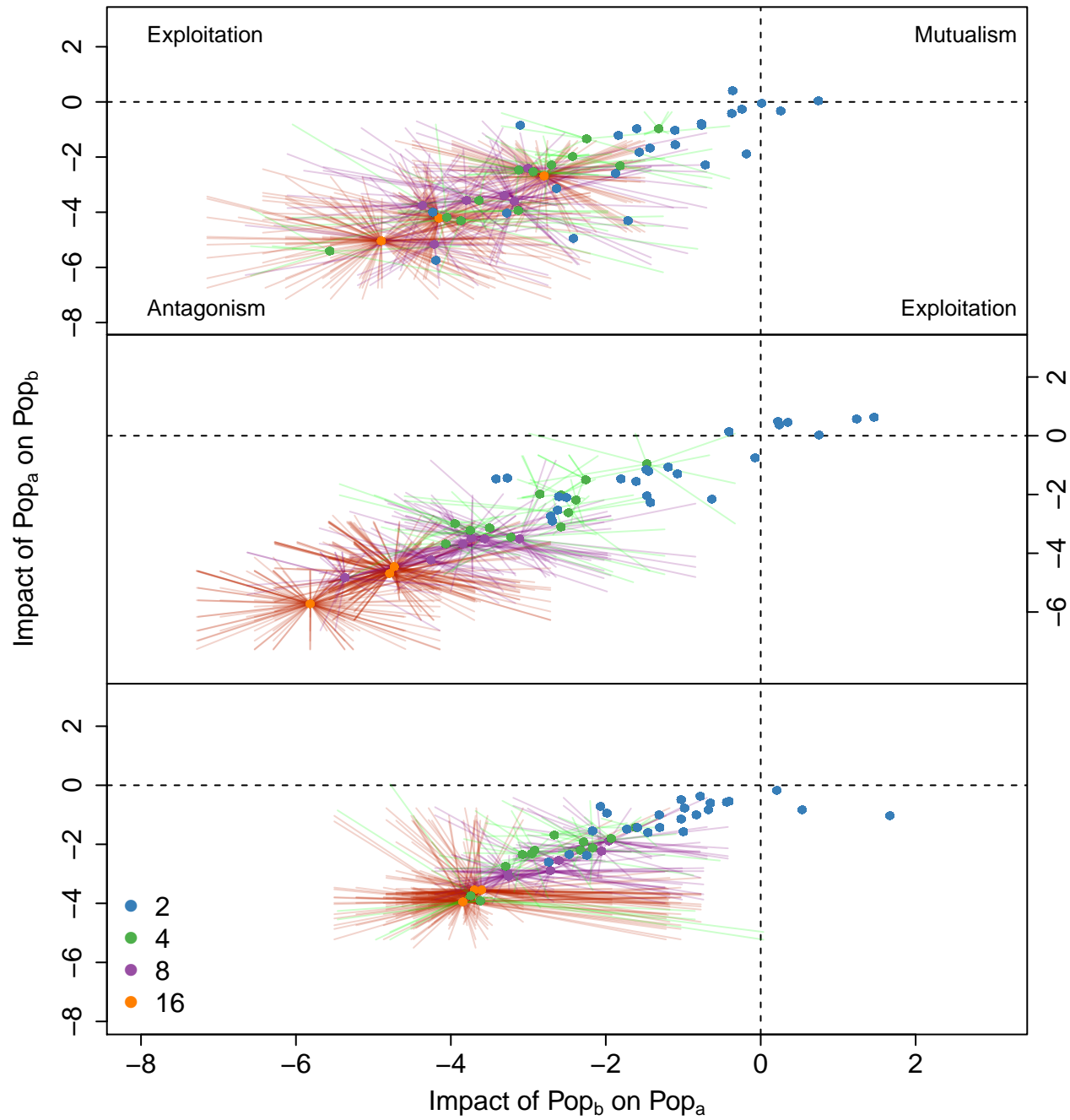

Supplement: Supplementary Figure S5 [file ismej201611x8.pdf]

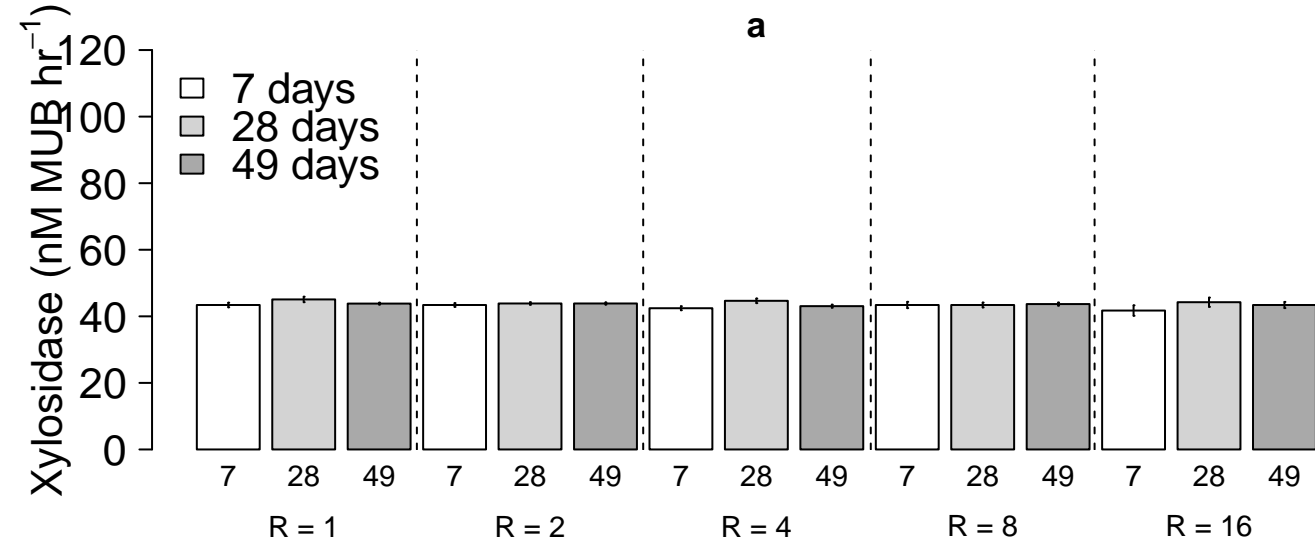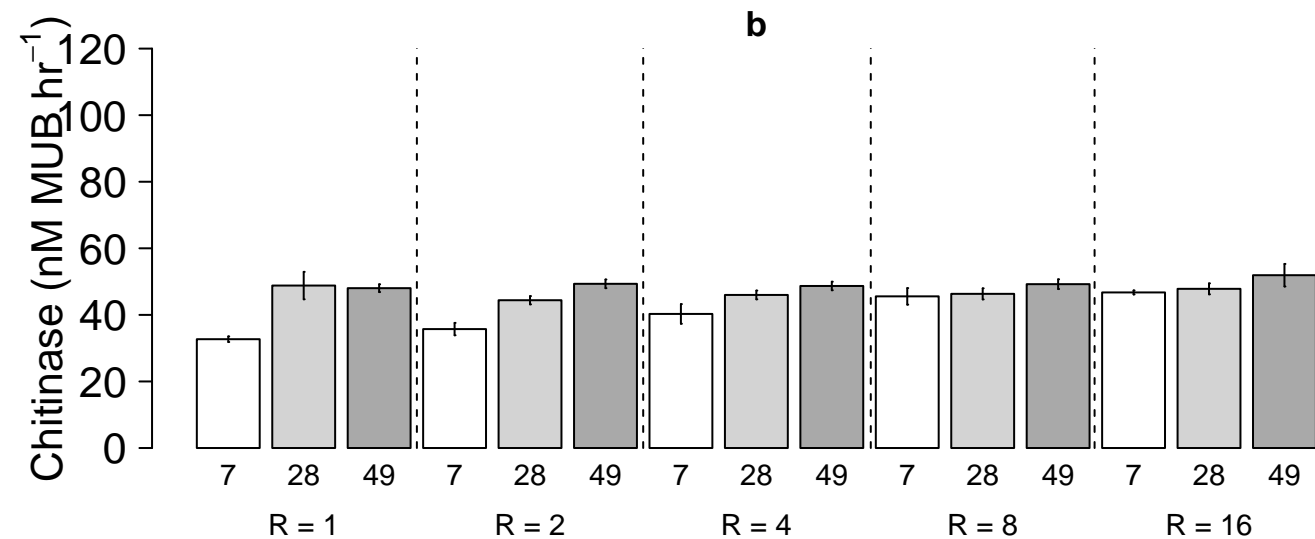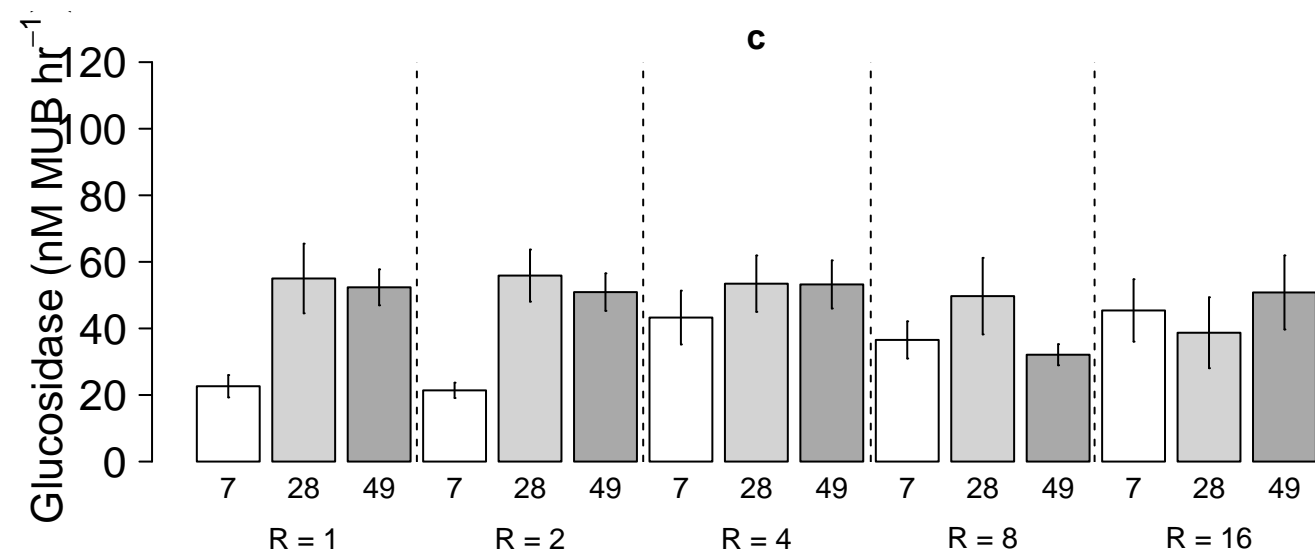

Supplement: Supplementary Figure S6 [file ismej201611x9.pdf]

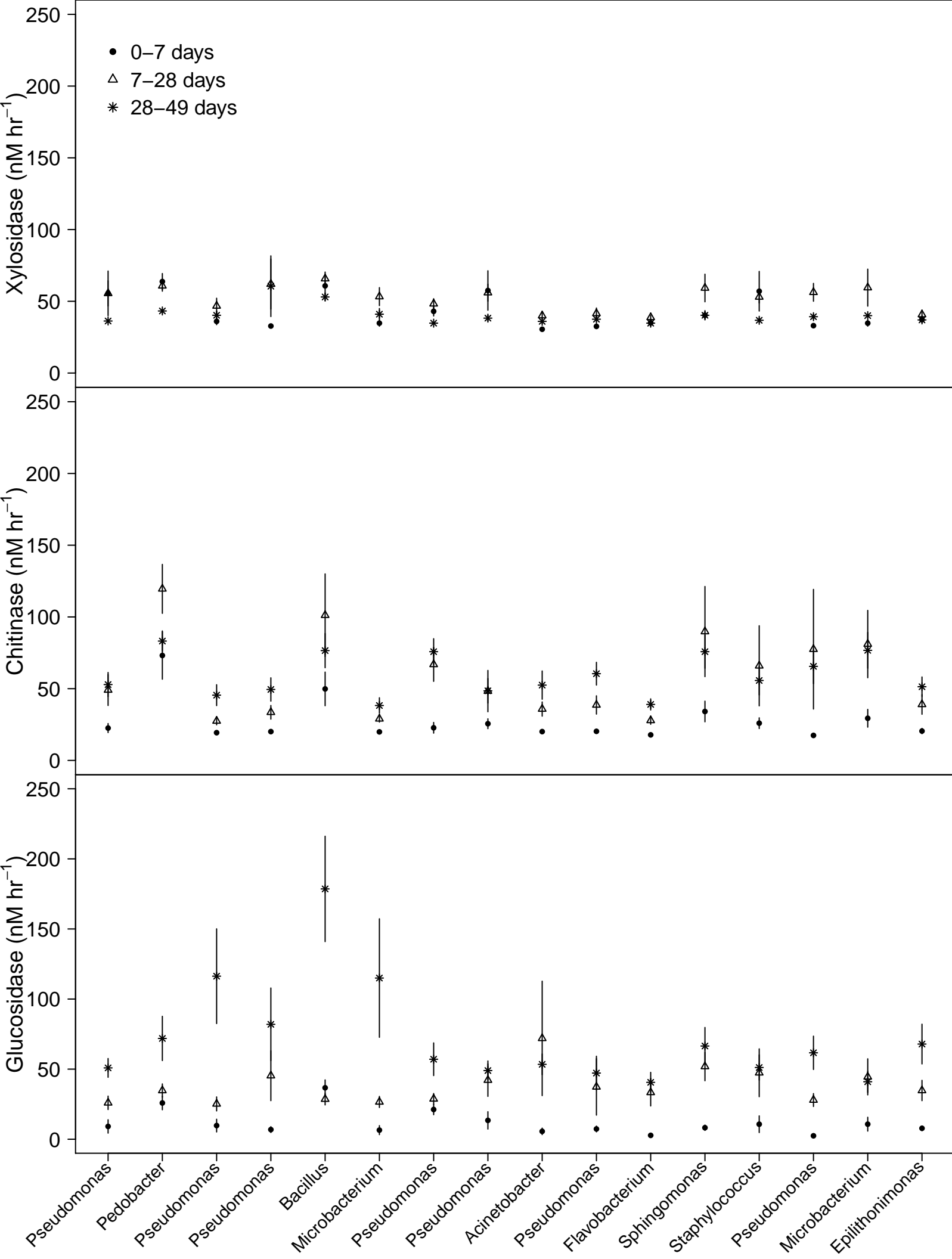

Supplement: Supplementary Figure S7 [file ismej201611x10.pdf]
